# Supplementary material for: Determinants of RNA metabolism in the Schizosaccharomyces pombe genome
Source: Mol Syst Biol. 2016 Feb 16;12(2):857. doi: 10.15252/msb.20156526 (PMC4770384; doi:10.15252/msb.20156526)
Supplement: Supplementary file 1 — Appendix [file MSB-12-857-s001.pdf]

Determinants of RNA metabolism in the *Schizosaccharomyces pombe* genome
-
Appendix

Philipp Eser*[1,2], Leonhard Wachutka*[2], Kerstin Maier[1], Carina Demel[1], Mariana Boroni[2], Srignanakshi Iyer[2], Patrick Cramer[1] and Julien Gagneur[2]

[1]Max-Planck-Institute for Biophysical Chemistry, Department of Molecular Biology, Am Fassberg 11, 37077 Göttingen, Germany
[2]Gene Center Munich and Department of Biochemistry, Center for Integrated Protein Science CIPSM, Ludwig-Maximilians-Universität München, Feodor-Lynen-Strasse 25, 81377 Munich, Germany

December 19, 2015

Contents

1 RNA-Seq read mapping **2**

2 Transcriptional unit mapping **2**

3 Estimation of RNA metabolism rates from 4tU-Seq data **3**
3.1 Overview . . . 3
3.2 Junction Model . . . 3
3.3 Exon model . . . 3
3.4 Uracil Bias . . . 4
3.5 Cross-contamination . . . 4
3.6 Expected number of reads given RNA species concentrations . . . 4
3.6.1 Expected number of reads . . . 4
3.6.2 Controlling for overall amount of labeled RNA and sequencing depth . . . 4
3.6.3 Controlling for TU length . . . 4
3.7 Parameter estimation . . . 5
3.7.1 Estimation of the dispersion parameter . . . 5
3.7.2 Overall estimation procedure . . . 6
3.8 Rescaling of synthesis rate . . . 6
3.9 Comparison of junction and exon model . . . 6
3.10 Note on incorporation lag time . . . 7

4 Identification of sequence elements predictive for rates and linear regression **7**

5 Validation of sequence model using Clément-Ziza *et al* (2014) eQTL dataset **8**
5.1 Read counts . . . 8
5.2 Fold change associated with local genetic variants . . . 8

6 Multivariate analysis of splicing time **9**

7 Appendix Figures **10**

1 RNA-Seq read mapping

Single- and paired-end RNA-Seq reads were mapped to the reference genome (ASM294v2.26) with the splice-aware aligner GSNAP Wu and Nacu (2010), excluding split reads and read pairs longer than 5000 nt, and allowing up to 7% mismatches. To detect known and novel splice sites, a splice site definition file compiled from the current annotation (Pombase V2.22) was supplied to GSNAP and the probabilistic model to identify splice junctions de novo (flag –novelsplicing) was used. After mapping, aligned paired-end reads were further filtered based on SAM flags contained in the alignment files to keep only pairs with proper pairing and orientation (-f 99, -f 147). Finally, PCR duplicates were removed with samtools Li *et al* (2009) rmdup (standard parameters). Splice sites were identified by using the CIGAR string of all mapped paired-end total RNA reads (replicates added). All sites supported by 10 or more spliced reads were considered for downstream analyzes. As only 48 introns were found with alternative splice sites (with identical start but different end coordinate, or vice-versa), alternative splicing was later on not considered. For each of those 48 splice sites, the splice junction with the highest read support was kept.

2 Transcriptional unit mapping

To map transcriptional units (TUs) we applied a segmentation algorithm to the paired-end Total RNA-Seq data separately for each strand. The per base coverage was extracted by considering the full fragments of a read-pair, e.g. from the start coordinate of the first read in the pair to the end coordinate of the second read in the pair. The cumulative read coverage vectors over the two biological replicate datasets was considered. The algorithm takes as input a coverage vector and three segmentation parameters: coverage cutoff, minimal length (min-length) and maximal gap (max-gap). First, all positions in the genome that exceeded the coverage cutoff were marked. Second, non-marked positions that were located between two marked positions that have a separation less than max-gap are also marked. Third, regions with a consecutive number of marked positions greater than min-length were reported. We used the current *S. pombe* annotation (Pombase V2.22) to estimate suitable values for the three segmentation parameters in our data: first, a coverage cutoff was estimated by an approach similar to David *et al* (2006). The distribution of the per base coverage between current annnotations was modeled as a bimodal distribution consisting in few non-annotated transcribed regions and a majority of non-transcribed (background) regions. The background-region distribution was modeled as a Gaussian distribution. The mode m of the background-region distribution was set to the median of the whole distribution. The variance of the background-region distribution was estimated as the variance of the distribution that is obtained by mirroring the part of the mixture with values lower than the mode m about the axis $y = m$. The cutoff (10.26) distinguishing background from transcribed regions was then set to a one-sided nominal p-value of 0.01 for the fitted Gaussian.

Second, min-length and max-gap are estimated simultaneously in an exhaustive search over all combinations of min-length and max-gap values between 10 and 1,000: for each combination, a segmentation of the genome was performed and scored based on the overlap with transcripts of the current annotation. We used the Jaccard index as a similarity measure, which is defined as the size of the intersection divided by the size of the union of the two sets. The Jaccard index reached maximal values for min-length and max-gap in the range between 50 and 500. Since there was no single optimal combination and the S. pombe transcriptome is very dense, we chose rather small parameters with min-length 200 and max-gap 80. With these parameters (coverage cutoff = 10.26, min-length = 200, max-gap = 80), the segmentation resulted in 7,062 TUs. To further improve this map of TUs, we only kept TUs that showed significant read coverage (average per base coverage < 20 in the two-minute labeled 4tU-Seq samples, normalized for sequencing depth using annotated ORFs read counts and following Love *et al* (2014). This filter resulted in a final set of 5,596 TUs.

3 Estimation of RNA metabolism rates from 4tU-Seq data

3.1 Overview

We used a probabilistic model that relates read counts of some kind (exonic reads, spliced and unspliced junction reads) to a set of model parameters $\mathbf{\Theta}$ which includes the RNA metabolism rates and technical nuisance parameters. With casual notations, we modeled the probability of observing read counts k of one kind in one sample as $p(k|\mathbf{\Theta}) = \mathrm{NB}(k|\text{mean}(\mathbf{\Theta}), \text{dispersion})$, where $\mathrm{NB}()$ is the negative binomial distribution. Subsections 3.2-3.5 model the RNA species concentrations in the sequenced samples and subsection 3.6 models the expected number of reads sequenced given these concentrations. This gives mean($\mathbf{\Theta}$). The last subsection describes the parameter estimation procedure.

3.2 Junction Model

For a given junction, let **[precursor RNA]** be the cellular concentration of the unspliced RNA and **[mature RNA]** the cellular concentration of the spliced RNA. With synthesis rate μ, splicing rate σ and degradation rate λ the following ODEs describe the dynamic of the system assuming first-order kinetics:

$$\begin{aligned}\frac{d\textbf{[precursor RNA]}}{dt} &= \mu - \sigma\textbf{[precursor RNA]}\\ \frac{d\textbf{[mature RNA]}}{dt} &= \sigma\textbf{[precursor RNA]} - \lambda\textbf{[mature RNA]}\end{aligned}$$

with following initial conditions:

$$\begin{aligned}\textbf{[precursor RNA]}_{\textbf{labeled}}|_{t=0} &= 0\\ \textbf{[mature RNA]}_{\textbf{labeled}}|_{t=0} &= 0\\ \textbf{[precursor RNA]}_{\textbf{unlabeled}}|_{t=0} &= \frac{\mu}{\sigma}\\ \textbf{[mature RNA]}_{\textbf{unlabeled}}|_{t=0} &= \frac{\mu}{\lambda}\end{aligned}$$

Under the assumption that introduction of labeled Uracils in the media ($t = 0$), the solutions are:

$$\begin{aligned}\textbf{[precursor RNA]}_{\textbf{labeled}}(t) &= \frac{\mu}{\sigma}(1 - e^{-\sigma t})\\ \textbf{[mature RNA]}_{\textbf{labeled}}(t) &= \frac{\mu}{\lambda(\lambda - \sigma)}(\lambda(1 - e^{-\sigma t}) - \sigma(1 - e^{-\lambda t}))\\ \textbf{[precursor RNA]}_{\textbf{unlabeled}}(t) &= \frac{\mu}{\sigma}e^{-\sigma t}\\ \textbf{[mature RNA]}_{\textbf{unlabeled}}(t) &= \frac{\mu}{\lambda(\lambda - \sigma)}(\lambda e^{-\sigma t} - \sigma e^{-\lambda t})\end{aligned}$$

3.3 Exon model

For single-exon TUs, there is no processing. Following the same rate notations, we obtain the same kinetics as for the precursor RNA (not that here λ plays the role σ earlier):

$$\begin{aligned}\textbf{[exon RNA]}_{\textbf{labeled}}(t) &= \frac{\mu}{\lambda}(1 - e^{-\lambda t})\\ \textbf{[exon RNA]}_{\textbf{unlabeled}}(t) &= \frac{\mu}{\lambda}e^{-\lambda t}\end{aligned}$$

3.4 Uracil Bias

Not all uracils available to the transcription machinery are labelled, leading to a labelling bias against transcripts with a small number of Us (Miller *et al*, 2011). Following Miller *et al* (2011), the probability $p(\text{4tUI})$ that one transcript incorporates at least one 4tU was modelled as:

$$p(\text{4tUI}) = 1 - (1 - p(\text{4tU replaces U}))^{\text{Number of U in transcript}}$$

This correction was difficult to apply to the junction model because of all possible RNA variants (isoforms, precursor and mature RNAs) overlapping the junction. However, we found that a U-bias correction would have negligible effects for intron-containing TUs because even their mature RNAs were containing generally containing many Us (short TUs were almost all single-exon). Hence, for typical values of $p(\text{4tU replaces U})$, $p(\text{4tUI})$ was very close to 1 for intron-containing TUs. In the following, U-bias correction was only applied to the exon model, which became:

$$\textbf{[exon RNA]}_{\textbf{labeled}}(t) = p(\text{4tUI})\frac{\mu}{\sigma}(1 - e^{-\sigma t})$$
$$\textbf{[exon RNA]}_{\textbf{unlabeled}}(t) = \frac{\mu}{\sigma}e^{-\sigma t} + (1 - p(\text{4tUI}))\frac{\mu}{\sigma}(1 - e^{-\sigma t})$$

3.5 Cross-contamination

What we measure is the purified and the not purified (so-called total) fractions of RNA. Measurements are sensitive to small amount of cross-contamination of unlabeled RNAs in the purified fraction, because unlabeled RNAs can represent the vast majority of RNAs especially at early time points. Thus, we introduced a cross-contamination factor χ that we assumed to be common to all RNA species for simplicity. Up to sample-specific factors common to all RNA species (see section 3.6), the concentration of purified and not purified RNA relates to the RNA cellular concentrations as:

$$\textbf{[purified RNA]} = (1 - \chi)\textbf{[labeled RNA]} + \chi\textbf{[unlabeled RNA]}$$
$$\textbf{[not purified RNA]} = \textbf{[labeled RNA]} + \textbf{[unlabeled RNA]}$$

3.6 Expected number of reads given RNA species concentrations

3.6.1 Expected number of reads

Let $x_{i,j}$ be the concentration of feature i in sample j (e.g. **[purified precursor RNA]** is the concentration of the feature 'unspliced read' in labeled samples). The expected counts $k_{i,j}$ of feature i in sample j was modeled as:

$$E(k_{i,j}) = F_j N_i x_{i,j}$$

where F_j is sample-specific scaling factor (see below) and N_i is the effective length of feature i (see below).

3.6.2 Controlling for overall amount of labeled RNA and sequencing depth

The RNA-sequencing protocol requires a constant amount of starting material and yields approximately the same number of reads per sample. Hence, the overall increase of labeled RNA over time is not reflected in the total amount of reads obtained. Therefore, normalization of the samples relative to each other had to be performed using sample-specific factors F_j. This normalization factor also allows controlling for variations in sequencing depth.

3.6.3 Controlling for TU length

The exon model is based on all reads overlapping the exon, and therefore depends on the exon length, yet not in a simple proportional fashion. Indeed, purified transcripts are sonicated into fragments of a typical length, in our case

about 200bp (mean fragment length, the actual number is not essential, it is only used in the derivation step). For asymptotically long transcripts, the expected number of fragments per transcript is:

$$N_i \approx \frac{\text{length of transcript } i}{\text{mean fragment length}}, \text{for } i \text{ long intronless TU}$$

However, this approximation fails for short transcripts. Indeed, sonication of short transcripts (about less than 2 times the mean fragment length) leads to a large fraction of very short fragments that are selected against during library preparation and do not get sequenced. Hence, to model the relation between fragment length and expected number of sequenced fragments for the whole range of transcript lengths, we empirically used a linear approximation that includes an offset L_{off} (see estimation below). This led us to an effective length such that:

$$N_i = \frac{\text{length of transcript } i + L_{\text{off}}}{\text{mean fragment length}}, \text{for } i \text{ intronless TU}$$

In contrast, the junction model relies on spliced and unspliced reads that overlap junctions. Reads that overlap a junction satisfy two criteria: i) they originate from fragments that overlap the junction and ii) the reads themselves overlap the junction. Junctions are typically further away from transcript ends compared to the fragment length. We therefore asssumed that the expected number of possible fragments satisfying criterion i) is the same for all junctions genome-wide. Criterion ii) implies that the effective length for the junction model is proportional to read length. Matching junction model and exon model estimates to the same scale was achieved by setting the effective length of the junction model to read length (78bp in our case) over mean fragment length:

$$N_i = \frac{\text{read length}}{\text{mean fragment length}}, \text{for } i \text{ spliced or unspliced junction}$$

Note that because the expected counts are linear in the effective length N_i, it is crucial to have a good estimate, otherwise the synthesis rates are not comparable between genes. This highlights an important advantage of the junction model over the exon model, since the former relies on fewer modeling assumptions with an effective length that is common to all junctions.

3.7 Parameter estimation

In the following we develop a method for estimating all parameters based on the observed count data by maximizing the likelihood.

Assuming negative binomial distribution of RNA-seq read counts (Love *et al*, 2014), the log likelihood reads as:

$$ll = \sum_{i,j} \log(\text{NB}(k_{i,j}|E_{i,j}(\Theta), \alpha) \quad (1)$$

where Θ is the set of parameters $\{\mu_i, \sigma_i, \lambda_i, F_j, \chi, L_{\text{off}}, p(4tUreplacesU)\}$ for all junctions or exons i and for all samples j, and where α is the dispersion parameter of the negative binomial. We assumed that the dispersion parameter is uniform over all samples and features, which we believe is a reasonable assumption.

3.7.1 Estimation of the dispersion parameter

Due to the complexity of the model and the large number of parameters it is not practically feasible to directly optimize the log likelihood. In a first step the mean $E_{i,j}$ of each data point (a data point is given by the number of reads belonging to one transcriptional feature e.g. exonic reads, junction reads at one time point) between the two replicates was computed. In a second step, the dispersion α was fitted by maximum likelihood letting the $E_{i,j}$ fixed:

$$\alpha = \text{argmax} \sum_{i,j} \log(\text{NB}(k_{i,j}|E_{i,j}, \alpha)) \quad (2)$$

Then the actual model was fitted using this value of α as fixed parameter. The expected counts obtained by this model were used again with (2) to get an improved estimate for α. Two rounds of iterations showed that α is a stable parameter and does not differ much from the first order guess (about 10% change). Forced changes of α by factor of 10 and 0.1 showed that the actual model parameters $\mathbf{\Theta}$ are quite robust against variation of α, since the estimated rates did not change significantly (relative changes 10^{-4}). Hence, we did not increase the number of iterations.

3.7.2 Overall estimation procedure

After extensive testing and numerical simulations, we found that the best results were obtained using the following procedure.

Transcripts with a length less than 120 base pairs were excluded from the analysis because of insufficient coverage, as the read length itself comprises 80 base pairs. We used the Broyden-Fletcher-Goldfarb-Shanno (BFGS) algorithm using the R function `optim()` with analytical gradient. We actually maximized the logarithm of the log likelihood, which turned out to give more reproducible results.

1. Start with $L_{\text{off}} = 0$, $p(4tUreplacesU) = 1$, and $\chi = 0$ and keep them as fixed parameters in the BFGS optimization procedure.

2. Select the 350 "best" intronless genes (in terms of coverage by visual inspection in a genome browser) and apply BFGS with the exon model (because we have the least number of assumptions here). The fitting is repeated 100 times using different start parameters, which allows us to estimate the robustness of the model. In this way we were able to extract the set of normalizing constants F_j for each sample (median of the fits). Because they are relative quantities, we deliberately set $F_1 = 1$.

3. Run the exon model and the junction model using the F_j as fixed input parameters. Each fit is done with 100 different initial values. Define the median of the calculated parameters as estimate.

4. Repeat step 2 and 3 using different values for the cross-contamination ranging from 0 to 5 % (independent experiments with spike-ins motivated this range), different L_{off} and $p(4tUreplacesU)$. These two parameters were set according to criteria described below.

Criteria for step 4 are as follows. Under the assumption that all junctions within one gene should have the same synthesis rate we chose the level of cross-contamination with the best correlation of the synthesis rates between the first and second junction of genes with 2 or more introns. When setting $p(4tUreplacesU)$ for the exon model so that these correlations match each other, we observed no significant correlations between synthesis rate and gene length. This result was in agreement with the junction model, for which no correlation between synthesis rate and gene length was found either. Moreover, the best value for $p(4tUreplacesU)$ was 1%, which is strictly within the expected range and close to the value of 0.5% estimated by (Miller *et al*, 2011) when profiling *S. cerevisiae* and in the range of estimates obtained by high performance liquid chromatography (Appendix Figure S2). Nonetheless, one should keep in mind that the lack of correlation between synthesis rate and gene length for intronless genes in our data is due to a modeling assumption and not a result of our investigations.

The results were used to improve our estimate of the dispersion parameter and steps 1 to 4 were repeated to improve the model parameters even further.

3.8 Rescaling of synthesis rate

The two quantities [purified RNA], [not purified RNA] are both linear in μ. Hence, the synthesis rate can only be estimated up to a global constant. We therefore arbitrarily set $F_1 = 1$ for the fitting. Absolute synthesis rates were then obtained by scaling all values so that the median steady-state expression level of ORF-TUs matches the one reported by a genome-wide absolute quantification study (median of 2.4 coding mRNAs per cell, Marguerat *et al* (2014)).

3.9 Comparison of junction and exon model

Synthesis and degradation rates within one TU showed higher consistency when estimated by the splice junction model than by the exon model (Spearman rank correlation = 0.44 versus 0.41 for synthesis time, Appendix Figure S3A,F,

and Spearman rank correlation = 0.79 versus 0.78 for half-life, Figures 3E and Appendix Figure S3G). The higher consistency of the junction model can be because i) it actually models the splicing step and ii) it is more robust against sequencing biases. We thus used rates estimated by the splice junction model for all intron-containing genes, and fell back to the exon model for intronless genes only.

3.10 Note on incorporation lag time

Our model did not consider time until 4tU gets available to the transcription machinery (by diffusion and import). The lag is a constant that is the same for all genes. Note that this time has to be very short since labeled RNA was detected after 2 min labeling.

4 Identification of sequence elements predictive for rates and linear regression

The goal of this procedure was to identify sequence elements predictive for a given rate of interest (synthesis, splicing, or degradation), in a given gene region of interest (promoter for all TUs and 5'UTR, coding sequence, introns, 3'UTR for ORF-TUs) and to estimate coefficients for each nucleotide at each position of these sequence elements. The procedure consisted of two consecutive stages 'seed finding' and 'seed extension and regression'.

The output of the 'seed finding' stage are initial sequence elements that associate with the rate. To this end, a linear mixed model was considered to assess the effect of each possible 6-mer in turn, while controlling for random effects over all 6-mers. We followed here an idea proposed by Liyang et al. (NAR, 2014) to estimate the activity of microRNAs. Formally, the effect of the j-th 6-mer on the rate was modeled according to:

$$\mathbf{y} = \mathbf{W}\boldsymbol{\alpha} + \mathbf{x}_j\beta_j + \mathbf{u} + \boldsymbol{\epsilon}$$
$$p(\mathbf{u}) = N(\mathbf{u}|0, \lambda\tau^{-1}\mathbf{K})$$
$$p(\boldsymbol{\epsilon}) = N(\boldsymbol{\epsilon}|0, \tau^{-1}\mathbf{I})$$

, where $\mathbf{y}$ is a n-vector of rates over all n TUs (respectively splice sites), $\mathbf{W}$ is an optional $n \times c$ matrix of covariates, $\boldsymbol{\alpha}$ is the corresponding vector of coefficients, $\mathbf{x}_j$ is th n-vector of the number of instances of the j-th 6-mer in the region over all TUs (respectively splice sites), β_j is its coefficient, $\mathbf{u}$ is a n-vector of random effects, and $\boldsymbol{\epsilon}$ is the n-vector of errors. For all rates, we considered as covariate the unit vector in order to model an intercept. We also considered as covariate the length of the 3'UTR in the case of the degradation rate, which we had found to be significantly associated with degradation. For other rates, no further covariate was used. The covariance matrix $\mathbf{K}$ was set to $\mathbf{X}^T\mathbf{X}$ where $\mathbf{X}$, whose columns are the $\mathbf{x}_j$, is the matrix of 6-mers counts. The covariance on the random effects allows controlling for the effects of all other 6-mers. The model was fitted using the GEMMA software (Zhou and Stephens, Nature Genetics, 2012). All 6-mers significantly associated with the rate (FDR <0.1, likelihood-ratio test with Benjamini-Hochberg correction for multiple testing) were retained. If both a 6-mer and its reverse complement were found significant, the two were considered as a single unstranded 6-mer, and the other ones as stranded 6-mers. Significant 6-mers overlapping by all but one or two base (eg. TTAATG and TAATGA) and sharing more than half of their genome-wide instances reciprocally were recursively assembled into longer k-mers (in this example TTAATGA). This procedure led to stranded and unstranded k-mers that we coined 'seed'.

The goal of the 'seed extension and regression' stage is to extend seeds to cover neighbor nucleotides significantly and to estimate the effect of each nucleotide. This is achieved with the following iterative procedure:

1. Initialization: The 'sites' are initialized by all elements in the region of interest matching the seed up to one mismatch (two mismaches for the long HOMOL-box motifs) together with 2 nucleotides 5' and 2 nucleotides 3' of it. For the unstranded motifs (two homol boxes) we also considered the reverse complements of the motifs as match.

2. Linear regression: We denote by n_i the number of sites for the i-th TU and L the length of a site. The 'consensus' sequence is defined as the sequence of the position-wise most frequent nucleotides over all sites. The following linear model is fitted by maximum likelihood:

$$y_i = \beta_0 + \sum_{j=1}^{n_i} \beta_{\text{cons}} + \sum_{k=1}^{L} \beta_{k,w_{i,j,k}} + \epsilon_i$$

where β_0 is the intercept, i.e. the average level in the absence of any site, β_{cons} is the effect of one consensus site, $w_{i,j,k}$ is the k-th nucleotide of the j-th site of the i-th TU, and $\beta_{k,A}$, $\beta_{k,C}$, $\beta_{k,G}$, $\beta_{k,T}$ are the effects of each nucleotide at position k relative to the nucleotide of the consensus site at the same position. By definition $\beta_{k,w}$ is constrained to be 0 if w equals the k-th nucleotide of the consensus sequence. The errors ϵ_i are assumed to be independently and identically normally distributed. Reverse complemented motifs enter in their canonical form.

3. Extension: For each site considered in step 2, its overall effect $\beta_{\text{cons}} + \sum_{k=1}^{L} \beta_{k,w_{i,j,k}}$ is tested to be significantly different from 0 ($P < 0.05$). To compute the p-value we evaluate the multivariate t-statistic (using glht of the multcomp package in R, see Hothorn et al. (2008) and Bretz et al. (2010)). A position weight matrix (PWM) is constructed based on all significant sites extended by 2 nucleotides 5' of the 5'-most significant position and 2 nucleotide 3' of the 3'-most significant position. To construct the PWM, the genomic nucleotide distribution is taken as background distribution. The sequences significantly matching the PWM (P> 0.80, multinomial model with a Dirichlet conjugate prior) are considered as the new sites. Step 2 and 3 are repeated until sites do not get extended in length. This is decided by visual inspection of the obtained PWM (the extended bases equal the background distribution). It turned out that the extension stage was only necessary and useful for the two well conserved Homol boxes.

The final motif sequence we report is the consensus sequence of the motifs. We searched again in the regions of interest for them (allowing 1 mismatch for all but the two HOMOL-Boxes (2 mismatches)). We finally applied the same linear model as in point 2. on these found sites to obtain the final coefficents.

5 Validation of sequence model using Clément-Ziza *et al* (2014) eQTL dataset

We compared fold-change associated with local genetic variants in Clément-Ziza *et al* (2014) with the predicted effects from the sequence-to-rate model described in the section 4.

5.1 Read counts

This study profiled steady-state RNA levels and not the newly synthesized RNAs. Hence, the coverage on introns was too poor to perform accurate quantification of the precursor RNAs. We thus focused on the quantification of steady-state levels of mature RNAs of our TUs. To this end, RNA-Seq data from recombinant *S. pombe* strains libraries (Clément-Ziza *et al*, 2014) were downloaded from ArrayExpress (`http://www.ebi.ac.uk/arrayexpress/`, identifier E-MTAB-2640). Genetic variants and strain genotypes was obtained as supplementary Datasets from the manuscript. RNA-Seq reads from each strain were mapped separately to the reference genome using STAR (version 2.4.0i) Dobin *et al* (2013) with default options. We considered for further analysis $k_{i,j}$, the number of reads overlapping at least one exon for TU i in sample j.

5.2 Fold change associated with local genetic variants

The read counts $k_{i,j}$ defined above were modelled according to the following generalized linear model:

$$\begin{aligned} k_{i,j} &\sim \text{NB}(\mu_{i,j}, \alpha_i) \\ \mu_{i,j} &= s_j \times q_{i,j} \\ \log_2(q_{i,j}) &= \beta_i^0 + \beta_i^{local} g_{i,j} + \sum_{b,batch} \beta_b^{batch} x_{j,b}^{batch} \end{aligned}$$

where NB is the negative binomial distribution, α_i is a gene-specific dispersion parameter; s_j is the size factor of sample j; $g_{i,j}$ is the genotype (0 for the reference allele, 1 for the alternative allele) at the variant of interest for gene i in sample j; $x_{j,b}^{batch}$ is 1 if sample j is from batch b and 0 otherwise. The model was implemented with the R/Bioconductor package DESeq2 (Love *et al*, 2014), which provides robust estimation of the size factors, of the dispersion parameters and the fold changes. The log-fold change of interest, β_i^{local}, together with its standard error, was then considered for further analysis. Effect of batches, reported in the original study, were dominating the signal and important to control for. We also investigated controlling for hotspots (8 eQTL hotspots were reported in the original study) but this led to an increased variance for little bias reduction.

6 Multivariate analysis of splicing time

We performed linear regression of log splicing time of each junction against i) all the nucleotides of 5'SS, the BS and the 3'SS region, ii) TU log-synthesis time, iii) the TU length and iv) the number of introns in the TU. The branchsite coordinates were predicted by the FELINES algorithm Drabenstot *et al* (2003). First regression was done against each covariate individually. Then, a joint model was built incrementally including each covariate in this order. Fraction of explained variance for both procedures are reported in table below.

Fraction of explained variance	Individual	Incremental
Sequence	0.50	0.50
log-synthesis time	0.45	0.42
TU length	0.09	0.07
Number of introns	0.034	0.0024

Except for the number of introns, all covariates contributed approximately equally in the individual and in the incremental model, showing that they are independently predicting synthesis time. In contrast, the number of introns did not added explained variance, likely because the predicted splicing time already correlated with number of introns (Compare Figure EV3F to Figure 6D). To determine the important nucleotides of the 5'SS, the BS and the 3'SS region, we used cross-validation. We started with all nucleotides +/-10 of the 5'SS, the BS and the 3'SS and decreased the window sizes systematically in several reduce steps. First we increased the starting position of the 5'SS -10 to -9,-8,-7, ... until cross-validation showed a loss in predictive power. Then we decreased the 5'SS +10 position in a similar manner. We continued analogously with the BS and 3'SS. We also used several different orders of removing the nucleotides (e.g. starting with 3'SS), to assert that we get similar results which are not biased by the order we apply the reduce step. We used 10-fold cross-validation. We trained the model on 9 parts and validated on the 10th part. Hereby we received a set of 10 models. To report the accuracy of our estimates we use the standard deviations of the coefficients reported by each model, the reported coefficients are the median of all 10 models.

7 Appendix Figures

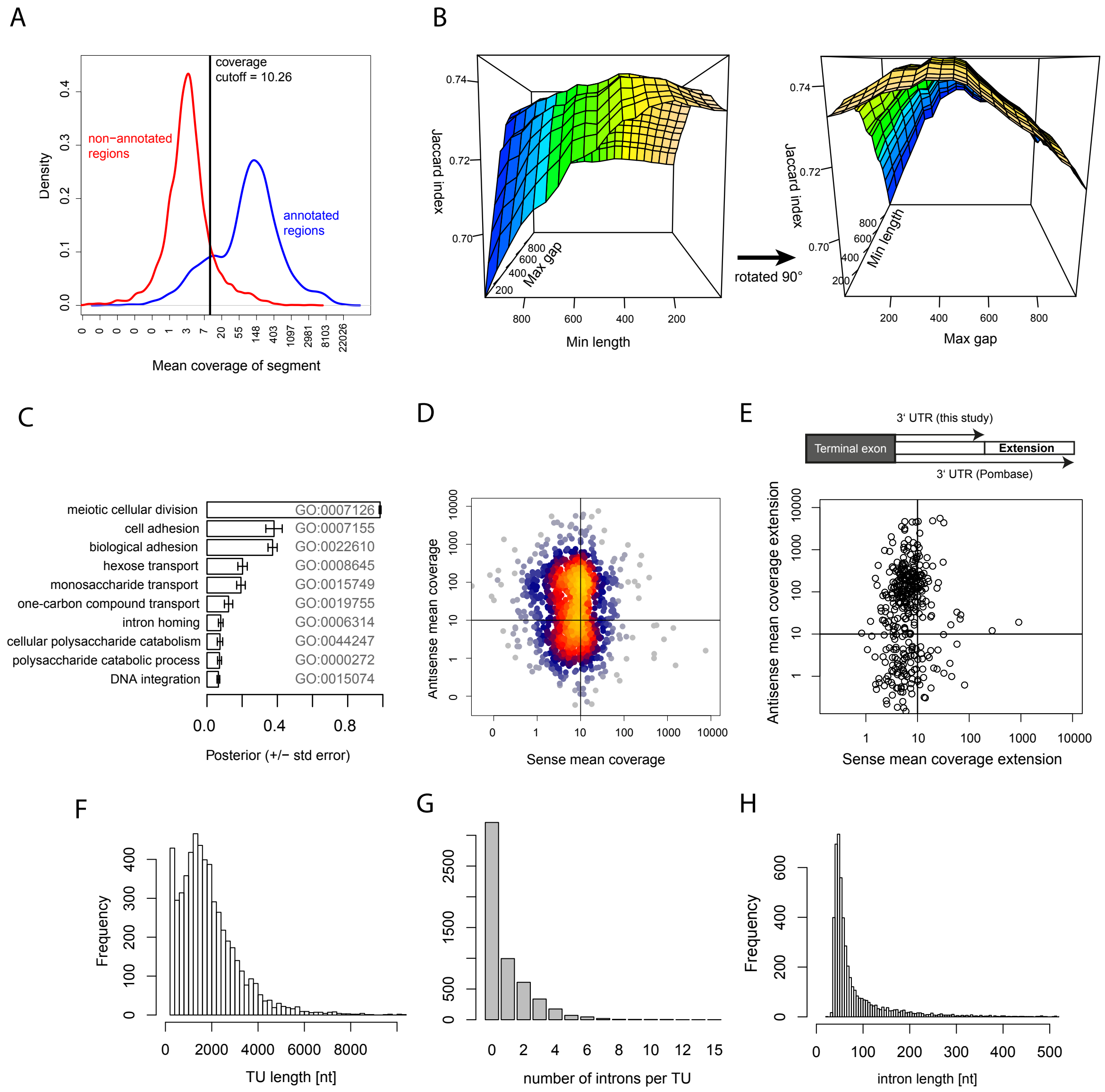

Appendix Figure S 1: **Segmentation algorithm parameters and antisense artifacts in current genome annotation.** (A) Distribution of mean RNA-Seq read coverage per segments for currently annotated (blue) and not currently annotated regions (red) and mean coverage cutoff for the segmentation algorithm to call a region expressed (vertical line). (B) Jaccard index (z-axis) when computing per base overlap between automatic segmentation and current annotation versus min-length and max-gap parameters of the segmentation algorithm. (C) Top ten GO terms enriched (model-based gene set analysis, Bauer et al. 2011) among 402 non-recovered protein coding genes from Pombase. (D) Sense mean coverage (x-axis) versus antisense mean coverage (y-axis) of 1011 non-recovered ncRNAs of the current annotation. (E) Mean sense coverage (x-axis) and antisense coverage (y-axis) of Pombase 3'UTR regions that extend TU defined 3'UTRs by 250nt or more. Per base coverage is extracted from total RNA-Seq data used in this study. The mass of the data in upper left quadrant indicate that long Pombase UTRs mostly arise from antisense artifacts in former studies. (F) Histogram of TU length. (G) Distribution of the number of introns per TU. (H) Histogram of intron length.

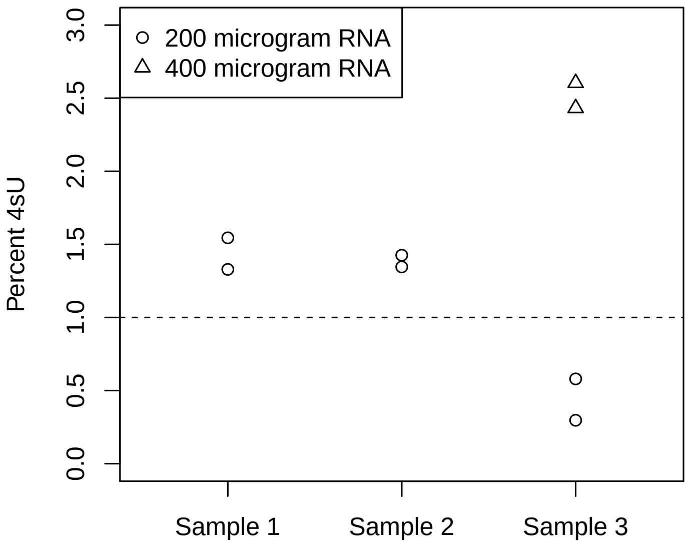

Appendix Figure S 2: **4tU incorporation probability by HPLC.** Proportion of 4sUs in RNA extracts of cells grown for 60 min. in 4tU media as estimated by HPLC (Materials and Methods) for 3 biological replicates (x-axis) using 200 microgram RNA (circle) and 400 microgram RNA (triangle), and estimate from 4tU-seq model (dashed horizontal line). Cells were grown for 60 min. to get rid of most RNAs present prior to 4tU exposure. HPLC 4sU peaks were measured because cells catabolize 4tU into 4sU prior to RNA incorporation.

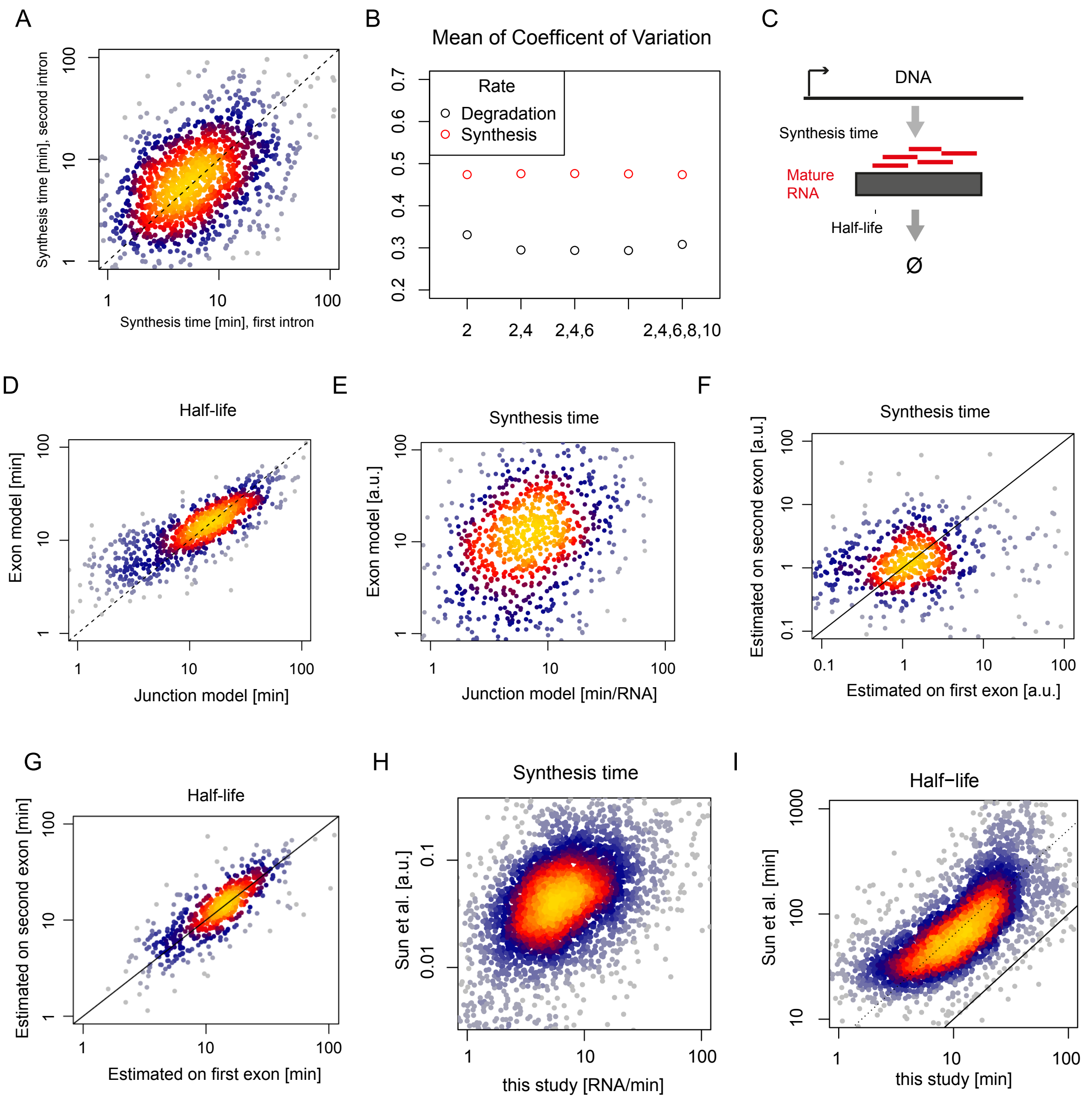

Appendix Figure S 3: **Modeling RNA kinetics.** (A) Estimate of synthesis time for ORF-TUs with two introns based on the first intron (x-axis) against estimate based on the second intron (y-axis). (B) Mean coefficient of variation for synthesis (red) and degradation (black) rates estimated from the two first introns of genes with two or more introns using total RNA-seq and the labeled RNA-seq at 2 min. only, 2 and 4 min., etc., up to the full series. (C) Exon-only model used to estimate synthesis times and half-lives of intronless TUs. (D) Half-lives of intron-containing TUs estimated using the junction model (x-axis) versus the exon model (y-axis). (E) As in (D) for synthesis times. (F) Synthesis time of intron-containing TUs estimated using the exon model on the first exon (x-axis) versus second exon (y-axis). (G) As in (F) for half-lives. (H) Comparison of synthesis times of ORF-TUs between this study (x-axis) against synthesis rates publised in (Sun et al. 2012). (I) As in (H) for half-lives.

A

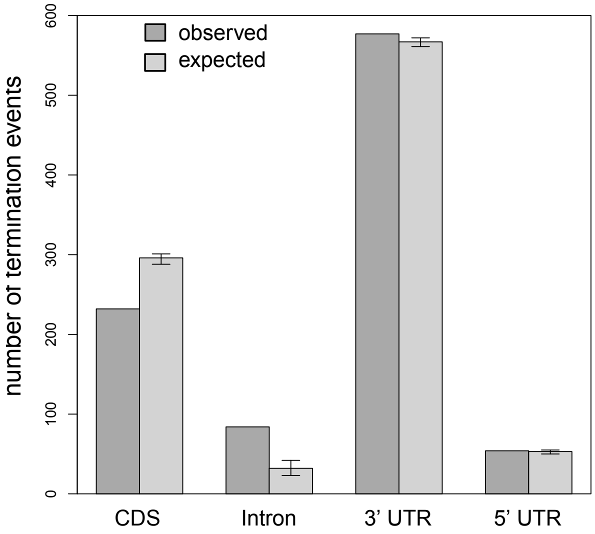

Appendix Figure S 4: **TU 3'-end in overlapping antisense ORF-TUs.** Observed and expected number of TU 3'-ends in CDS, intron, 5'UTR and 3'UTR of antisense ORF-TUs. Expected counts are estimated by 999 times randomization of all overlapping sense-antisense TU-pairs. Dark grey bars show the mean and the range which contains 90% of expected counts ("error bars").

References

Clément-Ziza M, Marsellach FX, Codlin S, Papadakis MA, Reinhardt S, Rodríguez-López M, Martin S, Marguerat S, Schmidt A, Lee E, Workman CT, Bähler J, Beyer A (2014) Natural genetic variation impacts expression levels of coding, non-coding, and antisense transcripts in fission yeast. *Molecular systems biology* **10**: 764

David L, Huber W, Granovskaia M, Toedling J, Palm CJ, Bofkin L, Jones T, Davis RW, Steinmetz LM (2006) A high-resolution map of transcription in the yeast genome. *PNAS* **103**: 5320–5325

Dobin A, Davis CA, Schlesinger F, Drenkow J, Zaleski C, Jha S, Batut P, Chaisson M, Gingeras TR (2013) STAR: ultrafast universal RNA-seq aligner. *Bioinformatics Oxford England* **29**: 15–21

Drabenstot SD, Kupfer DM, White JD, Dyer DW, Roe Ba, Buchanan KL, Murphy JW (2003) FELINES: a utility for extracting and examining EST-defined introns and exons. *Nucleic acids research* **31**: e141

Li H, Handsaker B, Wysoker A, Fennell T, Ruan J, Homer N, Marth G, Abecasis G, Durbin R (2009) The Sequence Alignment/Map format and SAMtools. *Bioinformatics* **25**: 2078–2079

Love MI, Huber W, Anders S (2014) Moderated estimation of fold change and dispersion for RNA-seq data with DESeq2. *Genome Biology* **15**: 550

Marguerat S, Lawler K, Brazma A, Bähler J (2014) Contributions of transcription and mRNA decay to gene expression dynamics of fission yeast in response to oxidative stress. *RNA Biology* **11**: 12

Miller C, Schwalb B, Maier K, Schulz D, Dümcke S, Zacher B, Mayer A, Sydow J, Marcinowski L, Dölken L, Martin DE, Tresch A, Cramer P (2011) Dynamic transcriptome analysis measures rates of mRNA synthesis and decay in yeast. *Molecular systems biology* **7**: 458

Wu TD, Nacu S (2010) Fast and SNP-tolerant detection of complex variants and splicing in short reads. *Bioinformatics* **26**: 873–881
