# Supplementary material for: Determinants of RNA metabolism in the Schizosaccharomyces pombe genome
Source: Mol Syst Biol. 2016 Feb 16;12(2):857. doi: 10.15252/msb.20156526 (PMC4770384; doi:10.15252/msb.20156526)
Supplement: Supplementary file 2 — Expanded View Figures PDF [file MSB-12-857-s002.pdf]

Expanded View Figures

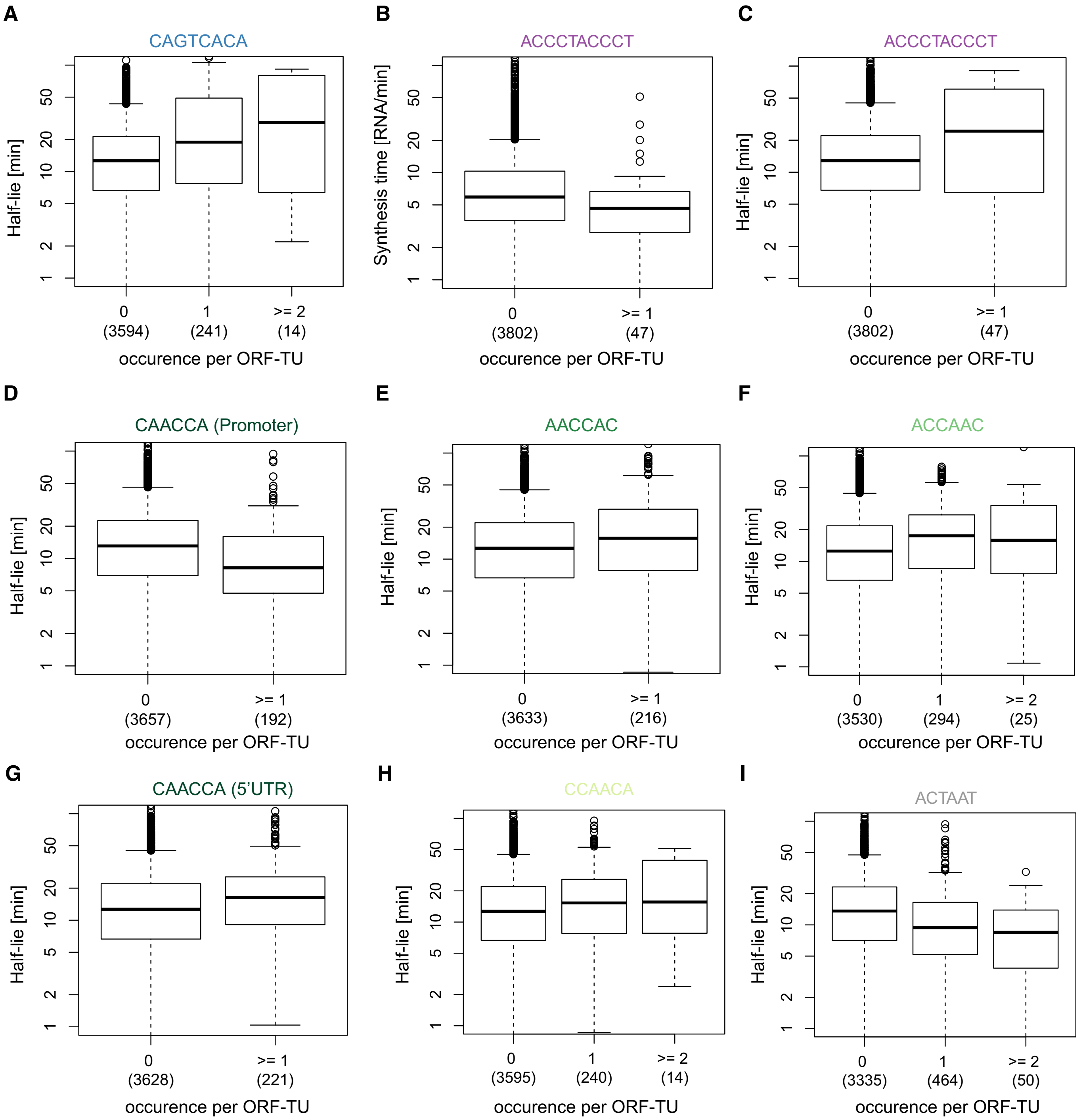

Figure EV1. Distribution of half-lives and synthesis times versus the number of of occurrences of identified motifs.

A Quartiles (boxes) and 1.5 times the interquartile range (whiskers) of the distribution showing the distribution of half-lives for all ORF-TUs, grouped by the number of occurrences of the motif CAGTCACA in their promoter sequence. Number of instances per box in parentheses.

B–I As in (A) for all identified motifs and corresponding rates.

© 2016 The Authors

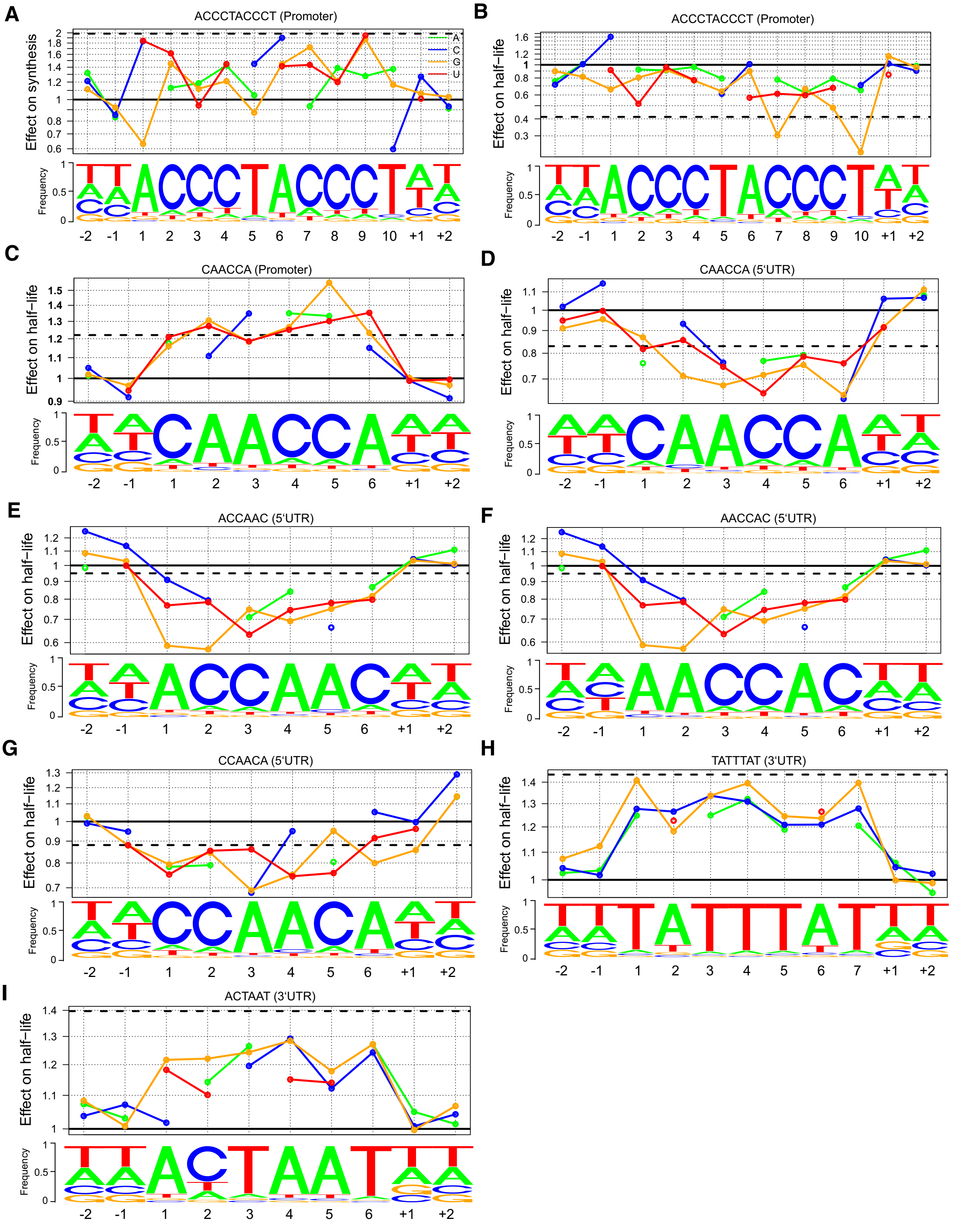

Figure EV2. Effect of single nucleotide substitutions.

A Nucleotide frequency within motif instances (lower track) and prediction of the relative effect on synthesis time (upper track) for single-nucleotide substitution in the Homol E-box consensus motif and of complete loss of the consensus motif (purple line).

B–I As in (A) for all identified motifs and corresponding rates.

© 2016 The Authors

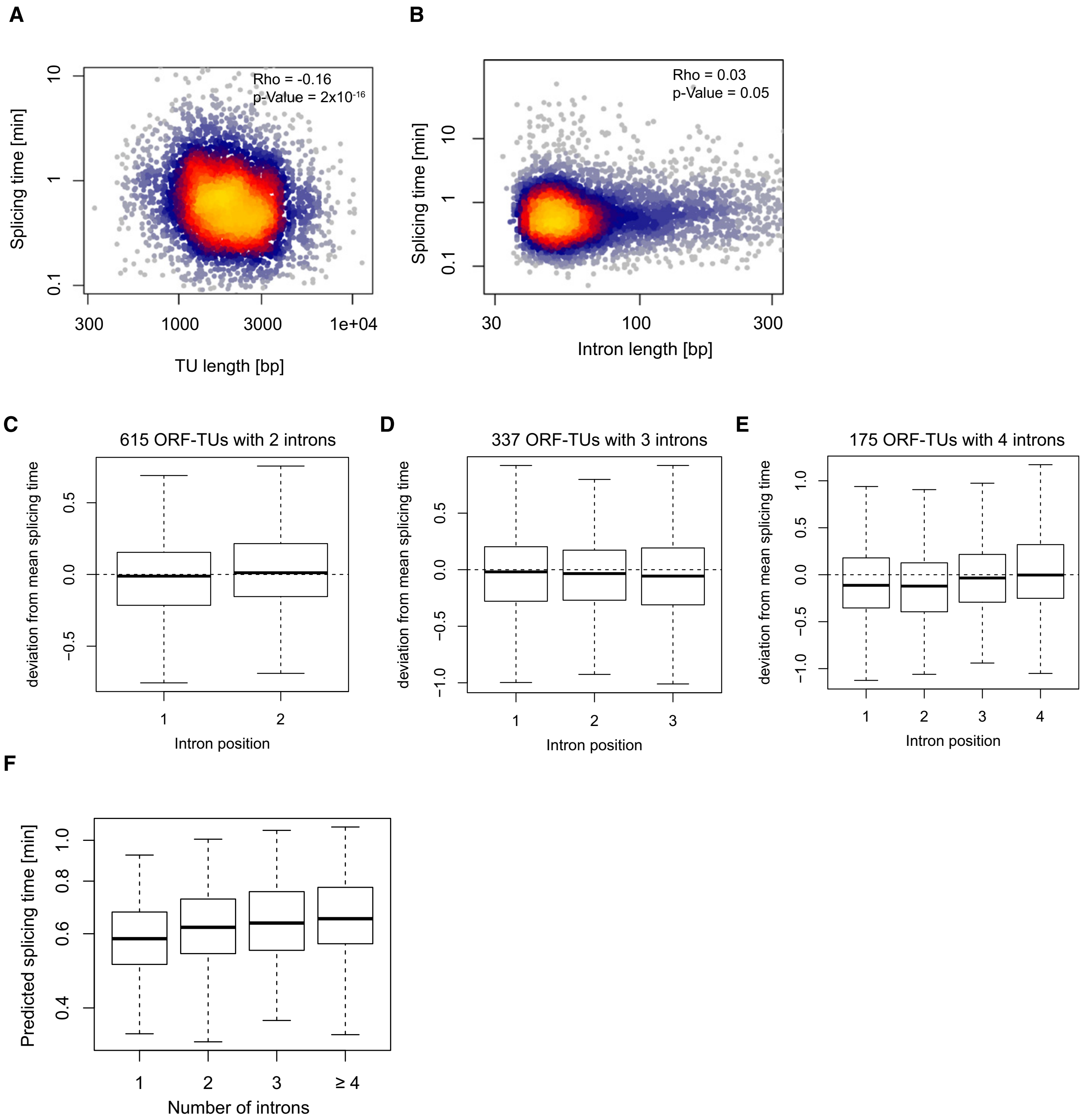

Figure EV3. Variables associating with splicing time.

A Mature mRNA length (*x*-axis) of all ORF-TUs with the splicing time (*y*-axis) of the corresponding introns.

B Intron length (*x*-axis) versus splicing time (*y*-axis) for all introns.

C–E Intron position-specific distributions of the deviation from the mean splicing time for ORF-TUs with two (C), three (D), or four (E) introns.

F Quartiles (boxes) and 1.5 times the interquartile range (whiskers) of the distribution of splicing time of individual introns predicted from their sequence at 5′SS, 3′SS, and BS (*y*-axis) versus the total number of introns in the corresponding ORF-TU (*x*-axis). This plot is to be compared to Fig 6D.

 © 2016 The Authors
